# A Novel High-Affinity Sucrose Transporter Is Required for Virulence of the Plant Pathogen *Ustilago maydis*

**DOI:** 10.1371/journal.pbio.1000303

**Published:** 2010-02-09

**Authors:** Ramon Wahl, Kathrin Wippel, Sarah Goos, Jörg Kämper, Norbert Sauer

**Affiliations:** 1 Karlsruhe Institute of Technology, Institute for Applied Biosciences, Department of Genetics, Karlsruhe, Germany; 2 Max-Planck-Institute for Terrestrial Microbiology, Marburg, Germany; 3 Friedrich-Alexander-University Erlangen-Nuremberg, Molecular Plant Physiology, Erlangen, Germany; Duke University Medical Center, United States of America

## Abstract

Plant pathogenic fungi cause massive yield losses and affect both quality and safety of food and feed produced from infected plants. The main objective of plant pathogenic fungi is to get access to the organic carbon sources of their carbon-autotrophic hosts. However, the chemical nature of the carbon source(s) and the mode of uptake are largely unknown. Here, we present a novel, plasma membrane-localized sucrose transporter (Srt1) from the corn smut fungus *Ustilago maydis* and its characterization as a fungal virulence factor. Srt1 has an unusually high substrate affinity, is absolutely sucrose specific, and allows the direct utilization of sucrose at the plant/fungal interface without extracellular hydrolysis and, thus, without the production of extracellular monosaccharides known to elicit plant immune responses. *srt1* is expressed exclusively during infection, and its deletion strongly reduces fungal virulence. This emphasizes the central role of this protein both for efficient carbon supply and for avoidance of apoplastic signals potentially recognized by the host.

## Abstract 2

A novel, high-affinity sucrose transporter identified in the plasma membrane of the plant pathogen *Ustilago maydis* is essential for fungal virulence and successful infection of maize.

## Author Summary

The plant parasitic fungus *Ustilago maydis* is a biotrophic pathogen that depends on live plant tissue for development. It is highly adapted to maize (*Zea mays*), where it causes the corn smut disease. Fungal cells growing within the plant apoplast are surrounded by the host plasma membrane at all growth stages, thereby establishing tight interaction zones with the host cells that assure optimal access to host-derived nutrients, including organic carbon sources. Here, we focus on the previously unknown feeding mechanisms of this plant pathogen within its host plant. We identified a fungal plasma membrane transporter, Srt1, that is expressed exclusively after plant infection and that turns out to be essential for virulence development of *Ustilago* in infected plants. Srt1 is the first characterized fungal transporter that allows direct utilization of sucrose without extracellular hydrolysis into monosaccharides, the carbon form more commonly taken up by pathogenic fungi. It is highly specific for sucrose, and its affinity largely exceeds that of equivalent plant transporters. This not only provides advantages for the carbon acquisition by the pathogen, but quite likely also offers a mechanism to prevent induction of plant defense responses known to occur upon apoplastic sucrose hydrolysis.

## Introduction

Plant pathogenic fungi cause major yield losses and affect the quality and safety of food and feed produced from infected plant material. Different fungi have developed different strategies to deal with their hosts. Infected plants are either kept alive to ensure a prolonged supply of organic carbon and other compounds to the pathogen (biotrophic fungi), or they are destroyed and the pathogen feeds on dead or dying plant tissue (necrotrophic fungi). Other fungi start with a biotrophic infection and switch to necrotrophic behavior at later stages of infection or under certain environmental conditions (hemibiotrophic fungi). Recognition of such pathogens by infected plants typically results in the production of reactive oxygen species and in hypersensitive cell death [1]. Obviously, plant defense responses resulting in hypersensitive cell death will be very effective against biotrophic fungi, whereas necrotrophic pathogens might even benefit from host cell death, and in fact, plants use different defense responses for biotrophic and necrotrophic fungi [1],[2]. The most important challenge for all pathogens is, therefore, the development of strategies allowing the avoidance of signals potentially recognized by the host.

**Figure 1:**
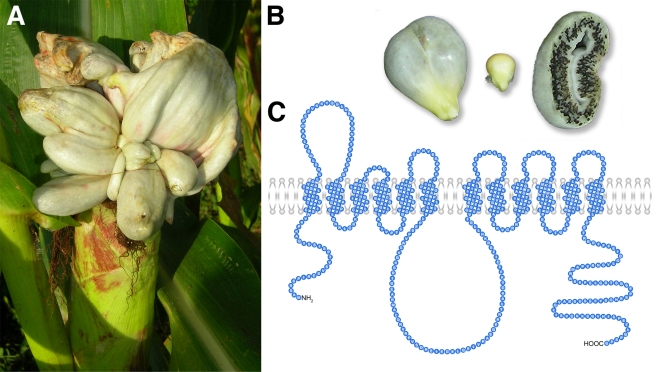
*U. maydis*–induced tumor formation in maize and predicted structure of Srt1. (A) Ear tumors of a maize plant infected with *U. maydis* that caused tumor induction. (B) Uninfected (middle) and *U. maydis*–infected, tumorous (left) maize kernels, plus a tumor section (right) showing layers of black fungal teliospores. (C) Putative topology of Srt1.

The basidiomycete *U. maydis* is a ubiquitous pathogen of maize (*Zea mays*), one of the world's most important cereal crops [3]. As a biotrophic fungus, *U. maydis* depends on living plant tissue and does not use aggressive virulence strategies [4]. During the infection process, fungal hyphae traverse plant cells without eliciting apparent host defense responses, a prerequisite for successful infection and persistent growth and development of a biotroph on its live host. *U. maydis* hyphae invaginate the plasma membranes of invaded plant cells, resulting in narrow contact zones that are perfectly suited for the uptake of organic carbon by the fungus [5]. Infections with *U. maydis* lead to the formation of tumors that consist of proliferating plant cells and of fungal hyphae (Figure 1A and 1B). Comparisons of transcript and metabolite levels in *U. maydis*-infected with noninfected maize leaves revealed an inhibition or delay in the sink-to-source transition of infected leaves [6],[7], which is in line with the increased carbon demand of the forming tumor.

All transport proteins identified so far in symbiotic or pathogenic fungus/plant interactions are specific for monosaccharides [8]–[10] and catalyze the uptake of glucose or fructose and, to a lesser extent, of other hexoses. It was speculated that these hexose transporters act in combination with fungal and/or plant-derived cell wall invertases [11],[12] to supply the pathogen with carbon derived from extracellular sucrose hydrolysis. The impact of these transporters on the development of fungal pathogens within the host plant has never been proven. However, plants have evolved mechanisms to sense extracellular (apoplastic) changes in glucose concentrations, e.g., produced from extracellular sucrose hydrolysis, and respond to these changes with the induction of defense responses [12]–[16]. Thus, feeding strategies avoiding invertase-derived glucose production in the apoplast might by advantageous especially for biotrophic fungi.

Here, we present the identification and functional characterization of Srt1, a novel high-affinity, sucrose-specific transporter from the biotrophic fungus *U. maydis*. We show that Srt1 represents a virulence factor essential for the successful development of the fungus within its host, as infections of maize with Δ*srt1* strains result in strongly reduced disease symptoms. The successful infection of maize by *U. maydis* without induction of defense responses is likely to result from an efficient competition of the *U. maydis* Srt1 protein with the low-affinity plant sucrose transporters for apoplastic sucrose, and potentially from the avoidance of apoplastic glucose signaling.

## Results

To address the relevance of sugar transporters for biotrophic development in *U. maydis*, we generated strains deleted for individual hexose transporters or hexose transporter-like proteins and assayed them for symptom development after syringe inoculation into young corn seedlings. Out of a total of 19 genes encoding hexose transporter-like proteins in the *U. maydis* genome (Figure S1 [17]), two were identified to influence the virulence of *U. maydis*. Here, we report the characterization of one of these genes (*um02374*, MIPS *Ustilago maydis* database, http://mips.helmholtz-muenchen.de/genre/proj/ustilago/) that was named *srt1* after the functional characterization of the encoded protein (Figure 1C) as a sucrose transporter.

### Deletion of *srt1* Reduces the Virulence of *U. maydis*, But Does Not Affect Plant Colonization or Fungal Growth on Axenic Media

**Figure 2:**
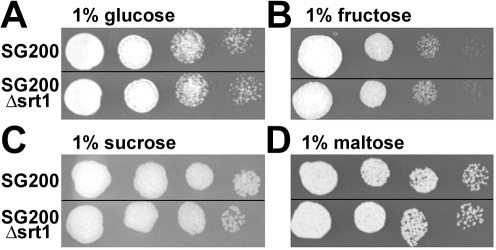
*srt1* deletion does not affect *U. maydis* growth in axenic culture. Growth of SG200Δ*srt1* on glutamine minimal media containing the monosaccharides (A) glucose or (B) fructose or the disaccharides (C) sucrose or (D) maltose is not reduced compared to the SG200 wild-type strain. Cultures from liquid glutamine minimal medium (1% glucose) were spotted in a series of 10-fold dilutions on the media indicated.

**Figure 3:**
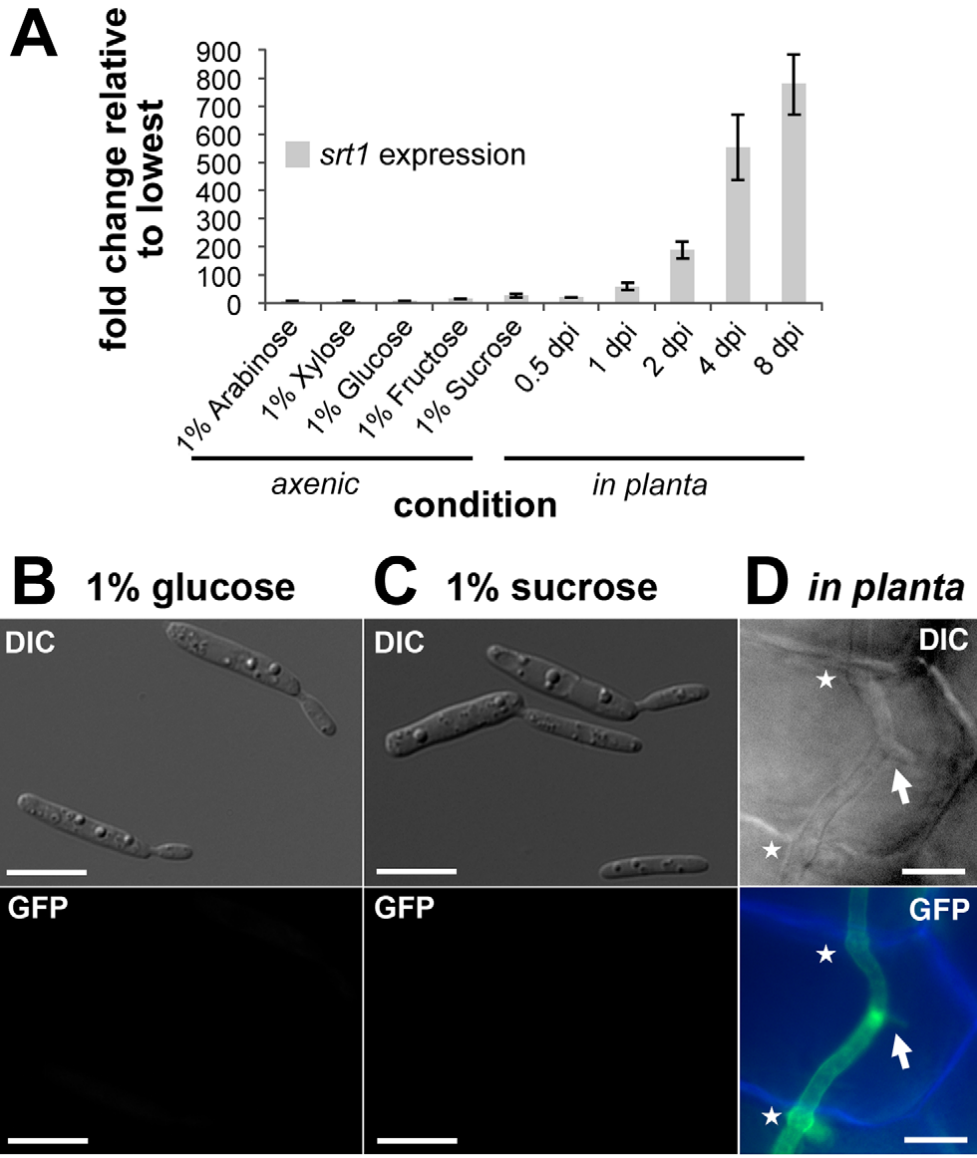
Srt1-GFP is specifically expressed in planta. (A) Expression profile (real-time PCR) of *srt1* in SG200 grown in liquid media supplemented with different carbon sources (left) or on plant tissue at different time points after infection. Gene expression was normalized to the expression of the constitutively expressed genes *actin* and *eIF2B*. Changes in *srt1* expression are displayed relative to the lowest expression value. (B) The SG200Δ*srt1*::*srt1*-*GFP* mutant shown to have a functional Srt1-GFP protein in Figure 3 was grown in minimal medium with 1% glucose. Cells were photographed in white light or under GFP excitation light (bottom). DIC, differential interference contrast microscopy. (C) SG200Δ*srt1*::*srt1*-*GFP* mutant photographed after growth in minimal medium with 1% sucrose. (D) In contrast to (B) and (C), hyphae of the SG200Δ*srt1*::*srt1*-*GFP* mutant show Srt1::GFP-derived fluorescence when monitored after infection of plant tissue (3 dpi). A DIC image (top) and two merged fluorescence images (blue indicates autofluorescence of plant cell walls; green, Srt1::GFP fluorescence of fungal hyphae) are shown. Arrows point towards clamp cells, which are formed by *U. maydis* only during in planta growth. Asterisks mark cell-to-cell penetration points. Bars represent 10 µm.

Compared to the progenitor strain SG200, a solopathogenic strain that can infect corn plants without a mating partner [17], *U. maydis* strains deleted for *srt1* (SG200Δ*srt1*) did not show altered growth on agar medium supplemented with different carbon sources (Figure 2A to 2D). This is in line with the observation that *srt1* expression is not detected under these conditions (Figure 3A). Moreover, the fact that *srt1* expression is not induced on medium without any carbon source demonstrates that it is not regulated by catabolite repression. In contrast, growth of wild-type *U. maydis* in planta results in a rapid induction of *srt1* expression (Figure 3A). Expression reaches a maximum at 4 to 8 days post infection (dpi) when most hyphae have reached the vascular bundles to spread inside the plant and when tumor formation is initiated. During earlier stages of infection only weak expression of *srt1* was observed (Figure 3A). This suggests that plant-derived signals are needed for *srt1* expression and that Srt1 might play a pivotal role in *U. maydis*/maize interaction.

These results were confirmed in analyses with a modified SG200 strain (SG200Δ*srt1*::*srt1*-*GFP*) in which the native *srt1* gene was replaced by an *srt1*-*GFP* fusion. Microscopic analysis of this strain revealed no fluorescence when cells were grown on minimal medium with 1% glucose (Figure 3B) or 1% sucrose (Figure 3C). After infection of maize leaves, however, a distinct GFP signal at the cell periphery was observed (Figure 3D). This (1) corroborates the plant-specific expression of *srt1* and (2) suggests a plasma membrane localization of the protein.

**Figure 4:**
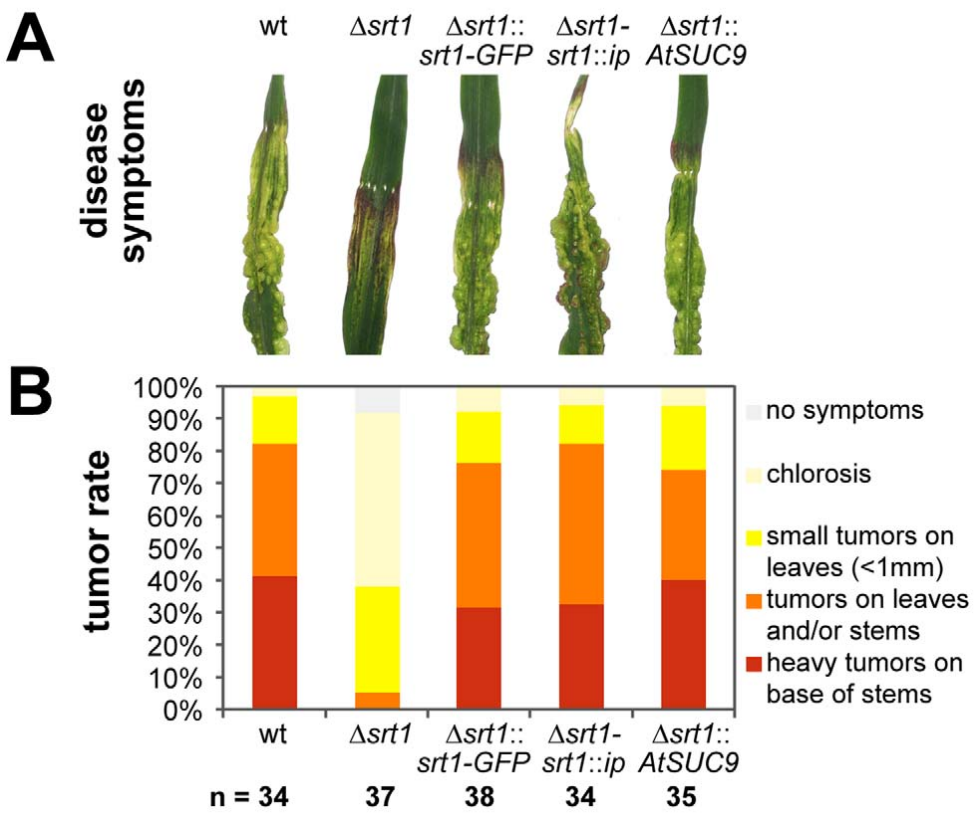
Srt1 is necessary for pathogenic development of *U. maydis*. (A) Tumor development at 7 dpi on maize leaves infected with the wild-type (wt) strain SG200, with an SG200Δ*srt1* deletion mutant, with a mutant strain that had its *str1* gene replaced by an *srt1*-*GFP* fusion construct under the control of the *srt1* promoter (SG200Δ*srt1*::*srt1*-*GFP*), with the Δ*srt1* deletion mutant complemented with a copy of *srt1* in the *ip* locus (SG200Δ*srt1*-*srt1*::*ip*), or with a mutant strain that had its *srt1* gene replaced by the *Arabidopsis AtSUC9* cDNA under the control of the *srt1* promoter (SG200Δ*srt1*::*AtSUC9*). (B) Disease rating at 7 dpi of plants infected with the wild-type strain (SG200), with three independent SG200Δ*srt1* mutants, with SG200Δ*srt1*::*srt1*-*GFP*, with three independently complemented SG200Δ*srt1*-*srt1*::*ip* strains, and with SG200Δ*srt1*::*AtSUC9*. Percentage and range of tumor formation of infected plants are color-coded (*n*  =  total number of plants analyzed). Error bars indicate the standard deviations of mean expression values.

Plant infection experiments with SG200 and SG200Δ*srt1* revealed major differences. Whereas infections with SG200 caused massive tumor formation (Figure 4A and 4B), infections with SG200Δ*srt1* resulted only in marginal disease symptoms. In most cases, infected plants showed no symptoms, only chlorotic lesions, or minute tumors (Figure 4A and 4B). Moreover, strain SG200Δ*srt1::srt1-GFP* which had been used for the analyses shown in Figure 3B to 3D displayed similar infection rates and symptom development as the wild-type strain, demonstrating that the *srt1*-*GFP* fusion encodes a functionally active Srt1-GFP protein.

To exclude the possibility that the observed loss of virulence in SG200Δ*srt1* mutants (Figure 4A and 4B) resulted from indirect effects and not from a loss of *srt1*, the *srt1* deletion mutant was complemented with an *srt1* wild-type copy. The resulting strain, SG200Δ*srt1*-*srt1*::*ip*, displayed similar infection rates and symptom development as SG200 or SG200Δ*srt1::srt1-GFP*. This confirmed that the observed reduced virulence of SG200Δ*srt1* mutant strains results from the loss of *srt1*.

With respect to tissue colonization, SG200Δ*srt1* hyphae did not differ from SG200 hyphae at the different developmental stages during disease progression (Figure S2).

### Srt1 Is an Energy-Dependent, Sucrose-Specific Transporter of the Fungal Plasma Membrane

The intronless *srt1* gene encodes a protein of 546 amino acids. The Srt1 protein has 12 predicted transmembrane domains (TMDs [18]) and a large extracellular loop between TMD1 and TMD2 (Figure 1C), a typical structural feature of previously characterized fungal and plant hexose transporters [8],[19]. Sequence comparisons revealed a moderate similarity (less than 30% identity) of Srt1 to a large group of transport proteins (Figure S3) that includes numerous well-characterized high-affinity monosaccharide transporters from plants and fungi as well as some low-affinity maltose transporters from *Saccharomyces cerevisiae* [20]–[22], *Pichia angusta* (synonym: *Hansenula polymorpha* [23]), or *Schizosaccharomyces pombe* [24]. Phylogenetic analyses revealed that Srt1 is most closely related to a small group of so-far uncharacterized proteins (Figure S3). This group contains uncharacterized transporters from different *Aspergillus* species (up to 47% identity) and from two biotrophic relatives of *U. maydis*, *Sporisorium reilianum* (88% identity) and *Ustilago hordei* (81% identity).

**Figure 5:**
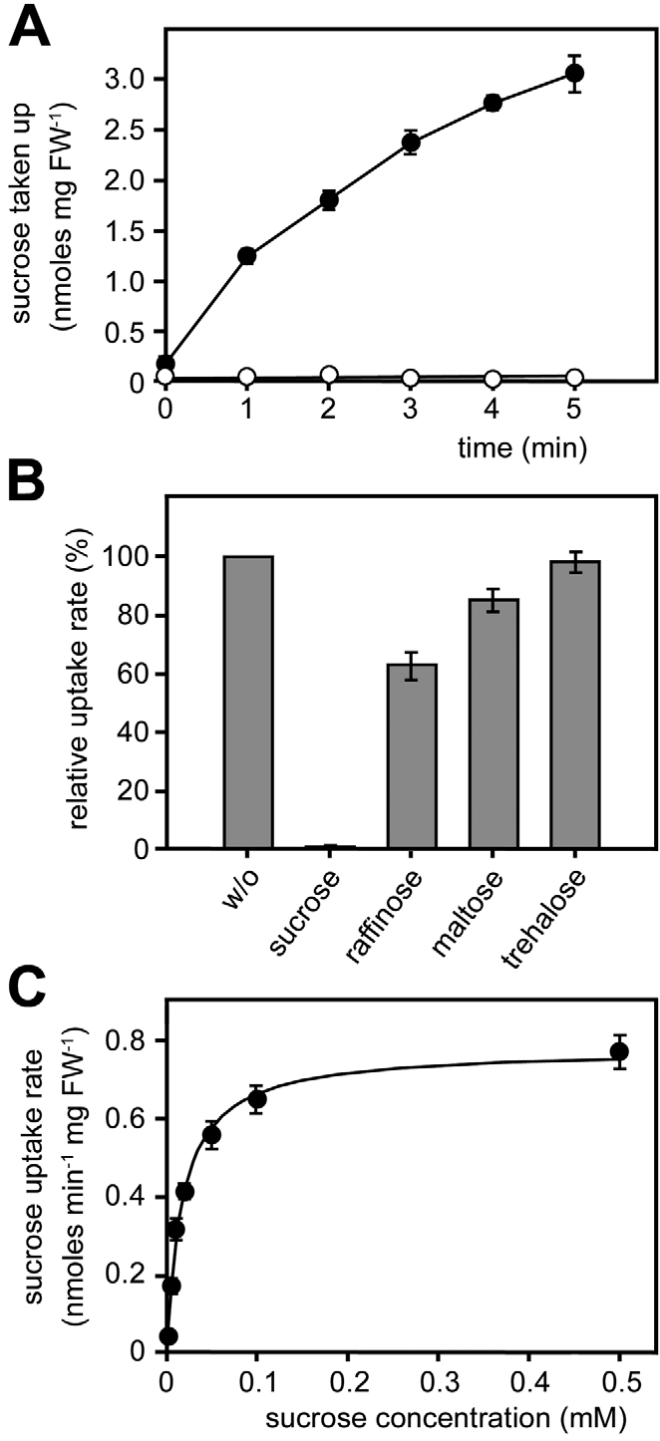
Srt1-dependent ^14^C-sucrose uptake in *S. cerevisiae*. (A) Uptake of ^14^C-sucrose by *srt1*-expressing (closed circles) and control cells (open circles). (B) Competition analysis (0.1 mM ^14^C-sucrose) with different potential substrates added at 100-fold molar excess. w/o, without. (C) Michaelis-Menten kinetics of sucrose uptake rates (pH 5.0) indicate a *K*_M_ of 26±4.3 µM (standard error [SE]). Error bars represent SE (*n* = 3).

To functionally characterize Srt1, the gene was expressed in the monosaccharide transport–deficient *S. cerevisiae* strain EBY.VW4000 [25], and uptake was analyzed with radiolabeled putative substrates (d-glucose, d-fructose, d-ribose, d-xylose, d-galactose, mannitol, sorbitol, xylitol, *myo*-inositol). As Srt1 did not catalyze the uptake of any of these compounds, additional tests were performed with ^14^C-sucrose and ^14^C-maltose. Because the *S. cerevisiae* strain EBY.VW4000 encodes an extracellular invertase that slowly hydrolyzes extracellular sucrose, these studies of Srt1 had to be performed in the invertase-deficient *S. cerevisiae* strain SEY2102 [26]. In fact, transport activity could be measured with ^14^C-sucrose (Figure 5A), but no uptake was observed for ^14^C-maltose (Figure S4). In competition analyses with an excess of unlabeled maltose (an alternative substrate of plant sucrose transporters), trehalose (an alternative substrate of *S. cerevisiae* maltose transporters), raffinose (an alternative substrate of the sucrose-hydrolyzing enzyme invertase), or sucrose (as positive control), raffinose was the only alternative compound that caused a minor inhibition of sucrose uptake (Figure 5B). No transporter described so far, not even the very well-characterized sucrose transporters from higher plants [27], showed such an extreme specificity for the disaccharide sucrose.

In fungi, sucrose transport activities were so far only described as side activities of broad-specificity, low-affinity maltose or maltotriose transporters [24],[28]. In uptake analyses in *S. cerevisiae* and with a wide range of different sucrose concentrations, the *K*_M_ of Srt1 for sucrose was found to be 26±4.3 µM (Figure 5C). Thus, the affinity of Srt1 for sucrose is several 100-fold to several 1,000-fold higher than that of the fungal maltose/maltotriose transporters [24],[28]. Moreover, its affinity is also much higher than that of higher plant sucrose transporters (20-fold to more than 200-fold), which catalyze sucrose uptake with *K*_M_ values in the millimolar range [23].

**Figure 6:**
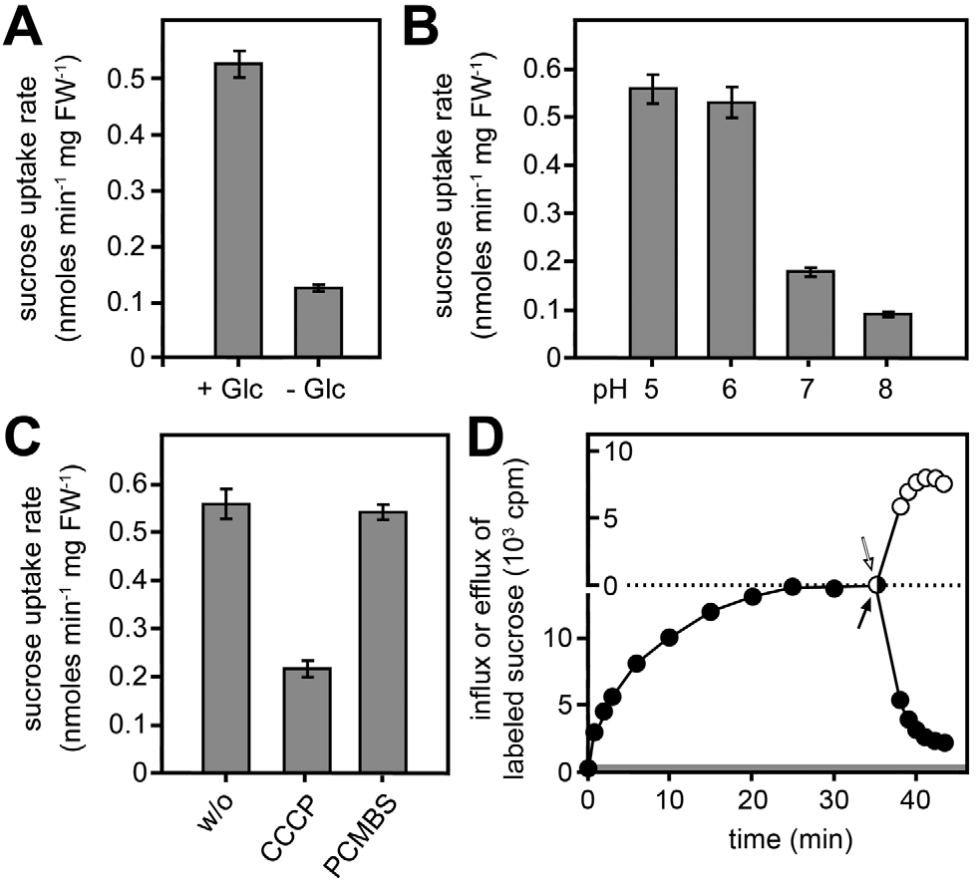
Transport characteristics of Srt1. (A) Transport is activated in the presence of the metabolizable carbon source glucose (Glc). (B) The pH optimum for sucrose uptake by Srt1 is in the acidic pH range. (C) Sucrose uptake is sensitive to the protonophore CCCP, but not to the SH-group inhibitor PCMBS. w/o, without. (D) The plateau of sucrose accumulation in baker's yeast results from an equilibrium of influx and efflux. Black symbols show the uptake of ^14^C-labeled sucrose and the onset of an immediate efflux, after replacement of labeled extracellular sucrose by unlabeled sucrose (black arrow). The grey region at the bottom of the graph shows the amount of sucrose that was sufficient to reach a concentration equilibrium of ^14^C-sucrose between the medium and the cell interior. White symbols show the onset of an immediate influx of ^14^C-labeled sucrose in an identical experiment that was started with unlabeled sucrose. The white arrow indicates the replacement of unlabeled extracellular sucrose by ^14^C-labeled sucrose. One of three experiments with identical results is presented. Error bars in (A) to (C) represent standard error (*n* = 3).

For the *S. cerevisiae* strain SEY2102, d-glucose represents the primary carbon source that can be both imported and metabolized. In contrast, sucrose can be imported when *srt1* is expressed, but it cannot be hydrolyzed due to a lack of invertase activity [26]. Therefore, if Srt1-mediated sucrose uptake is energy-dependent, the available energy might become limiting and the determined sucrose transport rates might be submaximal. In fact, the simultaneous presence of ^14^C-sucrose and glucose as metabolizable energy source strongly enhanced sucrose uptake (Figure 6A), which is indicative for an energy-dependent transport. In addition to this glucose-enhanced sucrose uptake, both the clear optimum of Srt1-driven sucrose transport at acidic pH values (Figure 6B) as well as the sensitivity to the protonophore carbonylcyanide *m*-chlorophenylhydrazone (CCCP; Figure 6C) underline that Srt1 is an active, energy-dependent H^+^-symporter.

These activities of plant sucrose transporters can be inhibited very specifically by the SH-group inhibitor *p*-chloro-mercuribenzene sulfonate (PCMBS) that does not affect plant hexose transporters [29]. In fact, the specificity of this inhibitor is so high that sucrose fluxes and phloem loading can be inhibited by PCMBS in whole plant or in intact plant tissues [30]. Srt1 is not inhibited by PCMBS (Figure 6C).

Expression of *srt1* in an *S. cerevisiae* strain (DBY2617) that possesses a cytoplasmic but no secreted invertase [31] enabled this strain not only to import ^14^C-sucrose, but also to grow efficiently on sucrose as sole carbon source (Figure S5). This proves that Srt1 activity alone is sufficient to meet the carbon import requirements of these cells. Thus, Srt1 is a high-affinity, high-capacity transporter that catalyzes the uptake of sufficient sucrose to fuel the growth of fungal cells.

**Figure 7:**
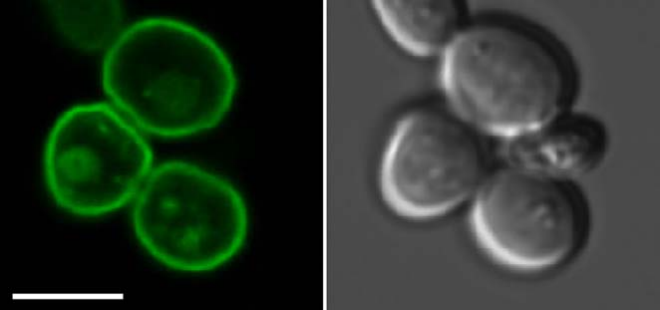
Subcellular localization of Srt1 in *S. cerevisiae*. A functional Srt1::GFP fusion protein localizes specifically to the plasma membranes of *S. cerevisiae*. The fusion construct was expressed under the control of the *S. cerevisiae pma1* promoter. The left image was taken under GFP excitation light; the corresponding image under transmission light is shown on the right side. The scale bar represents 5 µm.

Additional analyses of the subcellular localization in *S. cerevisiae* with a functional Srt1::GFP fusion protein demonstrated that, as expected from the transport assays (Figures 4 and 5) and complementation analysis (Figure S5), Srt1::GFP localizes exclusively to the plasma membrane (Figure 7).

### The *Arabidopsis* Sucrose Transporter AtSUC9 Can Functionally Replace Srt1

To validate that sucrose uptake is the primary function of Srt1 during biotrophic growth, we tested whether another transporter with a well-characterized sucrose uptake activity can functionally replace Srt1. We selected the sucrose transporter AtSUC9 from *Arabidopsis thaliana* [32]. This plant transporter is plasma membrane localized, transports sucrose and maltose, and is sensitive to CCCP and PCMBS. Moreover, AtSUC9 has a *K*_M_-sucrose of 0.5 mM [32], which is quite low for a plant sucrose transporter but still 20-fold higher than the *K*_M_-sucrose of Srt1 (Figure 5C). In strain SG200Δ*srt1*::*AtSUC9*, the *AtSUC9* cDNA was inserted into the *srt1* locus.

Figure 4 demonstrates that infections with SG200Δ*srt1*::*AtSUC9* are indistinguishable from wild-type infections with respect to tumor formation and frequency. Thus, the virulence of SG200Δ*srt1* can be restored by the expression of plant sucrose transporter cDNA *AtSUC9*.

## Discussion

The basidiomycete *U. maydis* is a biotrophic fungus that feeds on photoassimilated carbohydrates of maize to promote extensive proliferation within the plant tissue and within fungus-induced tumors (Figure 1). Deletion analyses of genes encoding hexose transporter-like proteins in *U. maydis* led to the identification of *srt1*. Under axenic growth conditions on different carbon sources, including sucrose (Figure 3A and 3C), this gene is not or only weakly expressed. Infection of maize tissue, however, causes a rapid induction of *srt1* expression (Figure 3A and 3D) that peaks at 4 to 8 dpi, when tumor formation is initiated. In agreement with these expression data, deletion of *srt1* affects neither axenic growth (Figure 2) nor the colonization of infected plants (Figure S2), but it results in strongly reduced symptom formation (Figure 4A and 4B).

Functional analyses in different *S. cerevisiae* strains characterized Srt1 as a plasma membrane-localized (Figure 7), energy-dependent (Figure 6A and 6C), high-affinity (Figure 5C) sucrose transporter with an unusually narrow substrate specificity (Figure 5B and Figure S4). *S. cerevisiae* cells expressing *srt1* do grow on sucrose as sole carbon source if they possess a cytoplasmic invertase (Figure S5), or they accumulate sucrose to high intracellular concentrations if this invertase is deleted (Figure 6D). This demonstrates that Srt1 is also a high-capacity transporter that can supply rapidly growing fungal cells with the carbon skeletons necessary for energy production and metabolism.

Fungal sucrose transporters with comparable kinetic properties and transport characteristics have so far not been cloned or characterized. *S. cerevisiae* has transporters that accept several α-glucosides, including maltose, trehalose, maltotriose, melezitose, α-methylglucoside, and sucrose. However, these transporters have *K*_M_ values for sucrose between 8 and 120 mM [28]. Moreover, transporters with *K*_M_ values in this concentration range have to compete with the *S. cerevisiae* extracellular invertase that hydrolyzes sucrose with a *K*_M_ that is also in the millimolar range.

In contrast to all of these transporters, Srt1 transports sucrose with high specificity and with an unusually low *K*_M_. The presented data demonstrate that the uptake of sucrose by Srt1 is not a possible side activity of this protein, but rather its only and exclusive function. They also show that Srt1 is a novel fungal sucrose transporter and that its activity is essential to develop full virulence of *U. maydis*.

### Srt1 Differs from Plant Sucrose Transporters in Two Functional Aspects

The primary physiological functions of plant sucrose transporters are the loading of sucrose into the phloem or the loading of sucrose into storage vacuoles, two processes that depend on the accumulation of high sucrose concentrations (up to 2 M) on one side of the respective membrane [27]. Uptake beyond a certain maximum is subject to feed back inhibition and total inactivation of sucrose transport. These activities of plant sucrose transporters can be inhibited very specifically by the SH-group inhibitor PCMBS that neither affects plant hexose transporters [29] nor Srt1 (Figure 6C). This is in accordance with the closer phylogenetic similarity of Srt1 to plant and fungal hexose transporters.

Srt1 is a transporter that imports sucrose for immediate consumption. Accumulation of high intracellular concentrations of sucrose in *U. maydis* is unlikely to occur. In invertase-deficient *srt1*-expressing *S. cerevisiae* cells, imported sucrose is not hydrolyzed, and Srt1 can, therefore, accumulate sucrose to concentrations higher than in the extracellular medium (more than 60-fold higher in Figure 6D). In contrast to plant sucrose transporters, the plateau of Srt1-mediated sucrose accumulation does not result from feed back (“shut-off”) inhibition of sucrose uptake, but rather from an equilibrium of sucrose influx and sucrose efflux, a typical property of transporters that do not accumulate their substrates under physiological conditions [33],[34].

In summary, Srt1 appears to be the prototype of a novel sucrose transporter that is unique with regards to its high specificity and its high affinity for sucrose, and that differs significantly in its functional behavior from sucrose transporters of higher plants.

### Srt1 Enables *U. maydis* to Feed on Apoplastic Sucrose without Extracellular Hydrolysis

**Figure 8:**
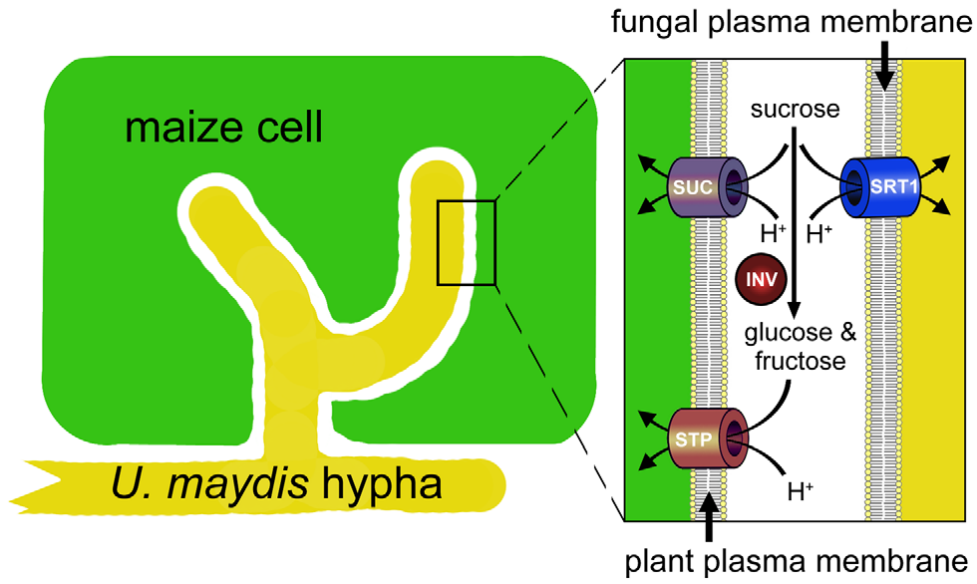
Model of the bidirectional competition for extracellular sucrose at the plant/fungus interface. Plants are known to use apoplastic sucrose either via plasma membrane-localized sucrose transporters (SUC or SUT proteins) or due to the activity of extracellular invertases (INV) via membrane-localized hexose transporters (STP or MST proteins). Srt1, a high-affinity sucrose H^+^-symporter, localizes to the fungal plasma membrane, and with its high substrate specificity and extremely low *K*_M_ value, it enables the fungus to efficiently use sucrose from the plant/fungus interface.

The primary long-distance transport and storage form of assimilated carbon in most higher plants, including maize, is sucrose. Apoplastic sucrose concentrations were determined in several dicot plants and are typically in the low-millimolar range [35]. Thus, a transporter with the properties of Srt1 represents a perfect tool for a biotrophic fungus that resides for a major part of its life cycle in the extracellular space of a living plant. The specificity and extremely high affinity of this transporter enables the pathogen to compete efficiently and successfully with the adjacent cells of its host for sucrose at the plant/fungus interface (Figure 8). Srt1 is perfectly suited to out-compete both the plants sucrose transporters (SUC or SUT proteins [27]) with their comparatively low substrate affinities as well as the invertase (INV)-dependent plant monosaccharide transporter (STP) proteins that are thought to feed different plant sink tissues (Figure 8) and that are known to be induced in response to elicitor treatment [36] or fungal infection [37]. Although most of STP proteins are high-affinity transporters, plant extracellular invertases have *K*_M_ values in the millimolar range and, therefore, seem to represent the rate-limiting step [38].

Under growth chamber conditions, an *U. maydis* mutant that had its *srt1* gene replaced by an *srt1* promoter/*AtSUC9* cDNA fusion showed wild-type virulence (Figure 4A). With a *K*_M_-sucrose of 0.5 mM [32], AtSUC9 has a lower substrate affinity than Srt1, but still one of the lowest *K*_M_-sucrose values determined for plant sucrose transporters. In contrast, the *K*_M_-sucrose of ZmSUT1, the sucrose transporter responsible for phloem loading in maize and, thus, the competing transporter at the *U. maydis*/maize interface, varies from 3.7 mM at pH 5.6 to 12.4 mM at pH 6.5 [39]. These different *K*_M_ values may explain the successful replacement of Srt1 by AtSUC9. Nevertheless, it could well be that SG200Δ*srt1*::*AtSUC9* would show reduced virulence in the field, where growth conditions are more competitive.

This result demonstrates that the primary function of Srt1 is, in deed, the supply of sucrose to the pathogen. Other possible functions, e.g., the signaling by interaction with a protein partner can be excluded, as it is highly unlikely that a foreign protein, such as AtSUC9, could complement such a function of Srt1.

Direct uptake of sucrose by a plant pathogenic fungus possibly provides also a second, more strategic advantage over the uptake of monosaccharides produced by the activity of a secreted fungal invertase. It was reported repeatedly that invertase-derived monosaccharides in the apoplast act as signaling molecules that trigger reduction of photosynthetic activity and induction of defense genes [13]–[16],[40]–[42]. Both responses are highly unfavorable for a biotrophic pathogen, as the first would reduce carbon availability for the pathogen and the second could even stop the infection. The use of a sucrose transporter rather than of an invertase/hexose transporter pair might, therefore, represent a mechanism of signal avoidance in an environment that is well prepared to sense and destroy potential pathogens.

The exclusive induction of *srt1* expression in tumor tissue implies that the transporter is specifically employed for sucrose uptake at the plant/fungal interface. During saprophytic growth on sucrose containing media the gene is neither expressed nor needed, since Δ*srt1* strains do not show reduced growth rates on media with sucrose as sole carbon source. As the presence of sucrose alone is not sufficient for *srt1* induction (Figure 3A and 3C), we must assume additional plant signals triggering the expression.

Srt1 allows direct utilization of apoplastic sucrose without prior hydrolysis in the extracellular lumen. During evolution of pathogenicity, especially of biotrophic fungi, this may have been a major step to successfully adapt to the hostile environment in host plants. The extremely high sucrose affinity and specificity of Srt1 not only has advantages for the carbon acquisition of the pathogen. It also offers a mechanism to prevent plant defense responses by avoiding the production of signaling molecules in the plant apoplast.

## Materials and Methods

### Strains and Growth Conditions

*Escherichia coli* strain TOP10 (Invitrogen) was used for cloning purposes. For plant infections, *U. maydis* cells were grown at 28°C in YEPSL [43]. For RNA extraction, *U. maydis* was grown in glutamine minimal medium, which is based an the minimal medium described by Holliday [44] with 30 mM l-glutamine as nitrogen source. Plant infections with *U. maydis* were performed as described [45]. The *U. maydis* strain used in this study is SG200, a haploid, solopathogenic strain that can infect maize plants without a mating partner [17]. *S. cerevisiae* strains used for analyses of Srt1 were EBY.VW4000 ([25] MATa; leu2-3,112; ura3-52; trp1-289; his3-Δ1; MAL2-8c; SUC2; Δhxt1-17; Δgal2; Δstl1; Δagt1; Δmph2; Δmph3), SEY2102 ([26] MATα; ura3-52; leu2-3,112; his4-519; suc2-Δ9; gal2), D458-1B ([46] MATα; leu2; itr1; ino1), and DBY2617 ([31] MATa; his4-539; lys2-801; ura3-52; suc2-438). Cells were grown in minimal medium (0.67% yeast nitrogen base without amino acids plus required amino acids depending on the strain) containing 2% maltose (EBY.VW4000) or glucose (all other strains) at 29°C.

SG200Δ*srt*: the deletion of *srt1* was performed by a PCR-based approach [47]. The promoter region of the *srt1* was amplified by PCR using primers 2374_LB1 (5′-TGG CTG TCA AGC CTC TTG AAG CAG-3′) and 2374_LB2 (5′-GAT GGC CGC GTT GGC CGC CAT GGT TAA GAG CAA GGG CGA C-3′), creating an *Sfi*I site at the 3′-end. The 3′ UTR sequence was amplified using primers 2374_RB1 (5′-CAC GGC CTG AGT GGC CAT CTC ACC TGA AAC TCT GCA GGC G-3′) and 2374_RB2 (5′-GCG TGC TCA TGT AGA CGG GAT AGC-3′, creating an SfiI site at the 5′-end. Both fragments were ligated to an SfiI Hyg^R^ fragment [47]. The entire *srt1* open reading frame (ORF) was replaced by a hygromycin resistance cassette in strain SG200.

SG200Δ*srt*::*srt1*-*GFP* was generated by fusing the ORF for eGFP to the 3′-end of the *srt1* ORF deleting the *srt1* stop codon. Primer pairs used to generate the flanks for homologous recombination were 2374_LB1Pf (5′-CGG GTC TCC CTT TCC TTC TTT TGC-3′) and 2374_LB2Pf (5′-GTT GGC CGC GTT GGC CGC TTG TGG ACT CGG CTG CAG AGT TTC-3′) for the flank matching the C-terminus of *srt1*, and 2374_RB1Pf (5′-GTT GGC CTG AGT GGC CTT GCA CTG CAC ATT CAC TAG CGG C-3′) and 2374_RB2 (5′-GCG TGC TCA TGT AGA CGG GAT AGC-3′) for the flank matching the 3′ UTR. Primers 2374_LB2Pf and 2374_RB1Pf carry the SfiI sites compatible to *eGFP* cassette of pUMA317 containing the hygromycin resistance gene [48]. The *eGFP* construct was integrated into the native *srt1* locus of SG200 by homologous recombination [47].

SG200Δ*srt*-*srt1*::*ip*: The Δ*srt1* deletion strain was complemented with the *srt1* gene under the control of its native promoter (about 2.5 kb of upstream sequence) three times independently by homologous recombination of pSRT1-GW into the ip-locus [43]. pSRT1-GW constructs were cloned according to the Gateway Cloning protocol (Invitrogen). attB-flanked PCR products of the 4.2 kb *srt1* locus were generated using primer pairs p2374_GW_for (5′-GGG GAC AAG TTT GTA CAA AAA AGC AGG CTG ACC ACC ATA AGT GCC ATT CTC GC-3′) and 2374Stop_GW_rev (5′-GGG GAC CAC TTT GTA CAA GAA AGC TGG GTT CAT TGT GGA CTC GGC TGC AGA GT-3′). BP and LR reactions were performed in one-tube format reaction using p123-BB-GW1 as destination vector. p123-BB-GW1 is a derivative of p123 [49], which was digested with the restriction enzymes HindIII and NotI, restriction sites were blunted using the Klenow polymerase, and the Reading Frame B Cassette was cloned into the plasmid backbone following the Gateway Vector Conversion System protocol.

SG200Δ*srt*::*AtSUC9*: Promoter and 3′ UTR sequences of *srt1* were amplified as described for the *srt1* deletion constructs. Both fragments were ligated upstream (promoter) and downstream (3′ UTR) of an SfiI 3xeGFP Hyg^R^ fragment of pUMA647 (K. Zarnack and M. Feldbrügge, unpublished data) in a derivative of the TOPO cloning vector (Invitrogen). A SfiI/AscI fragment containing the 3xeGFP ORF of pSRT3G was replaced by the AtSUC9 ORF that had been amplified with the primes AtSUC9c_SfiI_fwd (5′-GAG GCC AAC GCG GCC ACC ATG AGT GAC ATC CAA GCA AAA G-3′) and AtSUC9c_AscI_rev (5′-GGC GCG CCT TAA GGT AAA ACG GTA AGT GC-3′) that added *S*fiI and AscI cloning sites to the sequence. The resulting vector was pKW54. To exchange the *srt1* ORF of SG200 by *AtSUC9*, pKW54 was linearized with KpnI and integrated by homologous recombination into the *srt1* locus. The correct insertion was verified by Southern blot analysis of genomic DNA.

### DNA and RNA Procedures

Molecular methods followed described protocols [50]. DNA isolation from *U. maydis* and transformation procedures were performed as described [51]. Homologous integration of constructs was verified by gel blot analyses. Transformation of *S. cerevisiae* followed the protocol given in [52]. Total RNA from *U. maydis* cells grown in axenic culture was extracted using Trizol reagent (Invitrogen) according to the manufacturer's instructions. RNA samples to be used for real-time RT-PCR were further column purified (RNeasy; Qiagen) and the quality checked using a Bioanalyzer with an RNA 6000 Nano LabChip kit (Agilent).

### Cloning of *srt1* and Expression in *S. cerevisiae*

The *srt1* ORF was amplified from *U. maydis* genomic DNA using the primers 2374_EcoRI_for (5′-CAG AAT TCA AAA ATG GCG TCG TCT TCT CCC ATT CGT-3′) and 2374_EcoRI_rev (5′-CAG AAT TCT CGG ACT GCC AAG TCA TTG TGG AC-3′). DNA was sequenced and cloned into the *S. cerevisiae*/*E. coli* shuttle vector NEV-E [53], and the resulting plasmid was used for yeast transformation. For the fusion of Srt1 to the N-terminus of GFP, *srt1* ORF was PCR-amplified with primers that removed the stop codon. The resulting *srt1* ORF was cloned upstream of the ORF of GFP in the *S. cerevisiae* expression plasmid pEX-Tag [54].

### Transport Studies with Radiolabeled Substrates

*S. cerevisiae* cells were grown to an absorbance at 600 nm (A_600 nm_) of 1.0, harvested, washed twice with water, and resuspended in buffer to an A_600 nm_ of 10.0. If not otherwise indicated, uptake experiments were performed in 50 mM Na-phosphate buffer (pH 5.0) with an initial substrate concentration of 1 mM ^14^C-labeled sucrose (or another ^14^C-labeled or ^3^H-labeled substrate). Cells were shaken in a rotary shaker at 29°C, and transport tests were started by adding labeled substrate. Samples were withdrawn at given intervals, filtered on nitrocellulose filters (0.8-µm pore size), and washed with an excess of distilled H_2_O. Incorporation of radioactivity was determined by scintillation counting. Competition analyses were performed with 0.1 mM ^14^C-sucrose in the presence of 10 mM competitor (100-fold excess). For analyses of the energy dependence of sucrose transport, d-glucose was added to the yeast cells 2 min before the start of the experiment to a final concentration of 10 mM. For inhibitor analyses, CCCP (carbonylcyanide *m*-chlorophenylhydrazone) or PCMBS (*p*-chloromercuribencene sulfonate) were used at final concentrations of 50 µM.

For influx/efflux analyses in the plateau of sucrose accumulation (Figure 6D), identical amounts of *S. cerevisiae* cells were incubated in two flasks with either 100 µM ^14^C-labeled sucrose or with unlabeled sucrose, and sucrose uptake was determined in the flask with the labeled substrate. When the plateau was reached (after 35 min), the cells were quickly pelleted and washed in Na-phosphate buffer (pH. 5.0). Cells from the unlabeled flask were then resuspended to the initial volume with 100 µM ^14^C-sucrose, cells from the labeled flask with 100 µM unlabeled sucrose, and uptake experiments were continued.

### Light and Epifluorescence Microscopy

Light microscopic analyses were performed using a Zeiss Axioplan 2 microscope. Photomicrographs were obtained with an Axiocam HrM camera, and the images were processed with Axiovision (Zeiss) and Photoshop (Adobe). Chlorazole Black E staining of fungal cells in planta was performed as described [55]. GFP signals of Srt1::GFP (excitation at 450–490 nm, emission at 520 nm) in infected plant tissue or in sterile cultures, and autofluorescence of plant cell walls (excitation at 365 nm, emission at 397 nm) were visualized using an Axio Imager ZI microscope (Carl Zeiss). Images were processed with the AxioVision system (Carl Zeiss).

### Confocal Microscopy

Subcellular localization of the Srt1::GFP fusion protein in *S. cerevisiae* was determined by confocal microscopy (Leica TCS SPII; Leica Microsystems) and processed with the Leica Confocal Software 2.5 (Leica Microsystems). Emitted fluorescence was monitored at detection wavelengths longer than 510 nm.

### Quantitative Real-Time PCR Analysis

To analyze *srt1* expression on different carbon sources, SG200 was grown in glutamine minimal media supplemented with the indicated amount of the respective carbon source to an optical density at 600 nm (OD_600_) of 1.0 for 6 h. Precultures were grown overnight in glutamine minimal medium containing 1% of glucose. RNA samples were frozen in liquid nitrogen for two independently conducted replicates.

RNA of maize plants infected with SG200 was prepared as described [45].

Samples were taken 0.5, 1, 2, 4, and 8 dpi. For cDNA synthesis, the SuperScript III first-strand synthesis SuperMix assay (Invitrogen) was used on 1 µg of total RNA. qRT-PCR was performed on a Bio-Rad iCycler using the Platinum SYBR Green qPCR SuperMix-UDG (Invitrogen). The *U. maydis actin* (um11232) and *eIF2B* (um04869) genes were used as references. Primer sequences were rt-eIF-2B-F (5′-ATC CCG AAC AGC CCA AAC-3′) and rt-eIF-2B-R (5′-ATC GTC AAC CGC AAC CAC-3′) for *eIF2B*, rt-actin-F (5′-CAT GTA CGC CGG TAT CTC G-3′) and rt-actin-R (5′-CTC GGG AGG AGC AAC AAT C-3′) for the *actin* gene, and 2374_rt_for (5′-AGA CGC GTG GAA GGA CTT TCT TCG-3′) and 2374_rt_rev (5′-CCT AGC TCG AAC TTT GAC CAC CGC-3′) for *srt1*.

### Phylogenetic Analysis

For the phylogenetic analysis of the *U. maydis* Major Facilitator Superfamily (MFS) and for the identification of the 19 members of the *U. maydis* sugar transporter superfamily, 86 amino acid sequences of putative MFS members were obtained at MUMDB (IPR007114 Major facilitator superfamily; http://mips.helmholtz-muenchen.de/genre/proj/ustilago/). Two sequences of *U. maydis* ammonium transporters were included as out-group (Figure S1 and Table S1). For comparative phylogenetic analysis of Srt1, the amino acid sequence was aligned with 95 transporter sequences obtained by BLASTP analysis. This includes fungal and plant sequences with the highest similarity to Srt1, fungal and plant sequences with highest homology to *A. thaliana* sucrose transporters, as well as fungal and plant ammonium transporter sequences as out-group (Figure S3 and Table S2.). Sequences were aligned with MAFFT version 6 using the global alignment G-INS-i. A phylogenetic tree was calculated using the minimum linkage clustering method (http://align.bmr.kyushu-u.ac.jp/mafft/online/server/). TreeIllustrator 1.0.1 was used to visualize the Nexus formats of the MAFFT results.

## Supporting Information

Figure S1 **Phylogenetic analysis of the *U. maydis* Major Facilitator Superfamily.** Eighty-six amino acid sequences of putative Major Facilitator Superfamily (MFS) proteins were obtained at MUMDB (IPR007114 Major facilitator superfamily; http://mips.helmholtz-muenchen.de/genre/proj/ustilago/); two *U. maydis* ammonium transporter sequences were used as out-group (Table S1). The identified 19 members of the sugar transporter superfamily are highlighted as separated group within the tree. Phylogenetic distances of each branch are indicated as values.
(0.73 MB TIF)

Figure S2 **SG200Δ** ***srt1*** **hyphae do not differ with respect to leaf colonization from SG200 hyphae at 4 and 7 dpi during disease progression.** Chlorazole Black E staining of maize leaves infected with SG200Δ*srt1* and SG200 wild type at 4 and 7 dpi. (A) and (D) show hyphae of both strains at 4 dpi growing in the vicinity of a vascular bundle. (B) and (E) display hyphae at 7 dpi growing in the vicinity of a vascular bundle. (C) and (F) display collapsed hyphae that appear at 7 dpi in infections with both SG200 and SG200Δ*srt1*. (G) and (H) show an overview of a larger area infected with the two strains, respectively. In both cases, hyphae spread within the plant leave tissue. Scale bars indicate a magnification of 20 µm for (A), (B), (C), (D), (E), and (F) and 100 µm for (G) and (H).
(1.62 MB TIF)

Figure S3 **Comparative phylogenetic analyses of Srt1.** The Srt1 amino acid sequence was aligned with 117 transporter sequences obtained by BLASTP analysis. The analyses include fungal and plant sequences with the highest similarity to Srt1, fungal and plant sequences with highest homology to *A. thaliana* sucrose transporters, as well as fungal and plant ammonium transporter sequences as out-group (Table S2). A high phylogenetic distance is observed between the clade of potential plant and fungal sucrose transporters belonging to the Glycoside-Pentoside-Hexuronide Cation Symporter Family and the Srt1-like sucrose transporters belonging to the sugar transporter family. Species names, accession numbers, and where available gene names are given.
(0.91 MB TIF)

Figure S4 **^14^C-maltose is not a substrate for Srt1.** Uptake of ^14^C-maltose (closed circles) was determined in parallel with the uptake of ^14^C-sucrose (open circles) in the same *srt1*-expressing *S. cerevisiae* cells that had been used to determine transport in Figure 4. The extracellular pH was 5.0, substrate concentration was 1 mM. Although ^14^C-maltose transport was analyzed for much longer than the transport of ^14^C-sucrose (see also Figure 4), no significant import of ^14^C-maltose into *srt1*-expressing cells could be observed. Error bars represent standard error (*n* = 3).
(0.07 MB TIF)

Figure S5 **Srt1 complements the growth defect of** ***S. cerevisiae*** **strain DBY2617.** DBY2617 possesses a cytoplasmic invertase, but lacks an extracellular invertase and a sucrose transport activity. Therefore, it cannot use extracellular sucrose as carbon source. Transformation with a plasmid that drives expression of *srt1* complements this defect and allows growth on sucrose as sole carbon source. Transformation with the empty vector (NEV-E) allows only limited growth that is due to passive diffusion of sucrose into the cells.
(0.43 MB TIF)

Table S1 ***U. maydis*** **transporter proteins of the Major Facilitator Superfamily.** Accession number, gene number (MUMDB [IPR007114 Major facilitator superfamily; http://mips.helmholtz-muenchen.de/genre/proj/ustilago/) and predicted function of the putative transport proteins used to calculate the phylogenetic tree shown in Figure S1.
(0.04 MB DOC)

Table S2 **Proteins used for comparative phylogenetic analyses of Srt1.** Accession numbers, putative or determined functions of the transport proteins used to calculate the phylogenetic tree shown in Figure S3.
(0.06 MB DOC)

## Abbreviations

dpi: days post infection
