# Supplementary figures and images for: A Novel High-Affinity Sucrose Transporter Is Required for Virulence of the Plant Pathogen Ustilago maydis
Source: PLoS Biol. 2010 Feb 9;8(2):e1000303. doi: 10.1371/journal.pbio.1000303 (PMC2817709; doi:10.1371/journal.pbio.1000303)

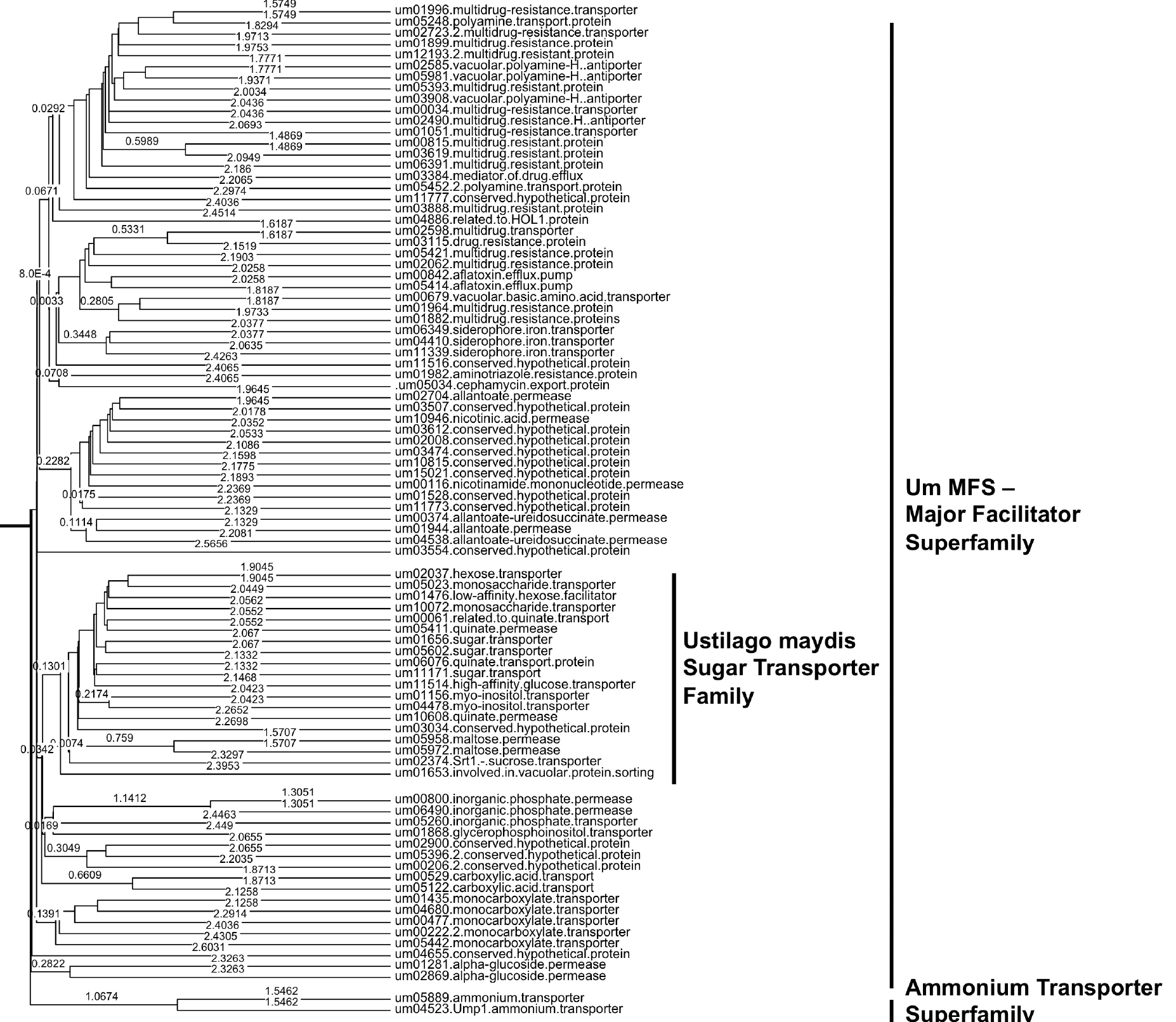

Supplement: Figure S1 — Phylogenetic analysis of the U. maydis Major Facilitator Superfamily. Eighty-six amino acid sequences of putative Major Facilitator Superfamily (MFS) proteins were obtained at MUMDB (IPR007114 Major facilitator superfamily; http://mips.helmholtz-muenchen.de/genre/proj/ustilago/); two U. maydis ammonium transporter sequences were used as out-group (Table S1). The identified 19 members of the sugar transporter superfamily are highlighted as separated group within the tree. Phylogenetic distances of each branch are indicated as values. (0.73 MB TIF) [file pbio.1000303.s001.tif]

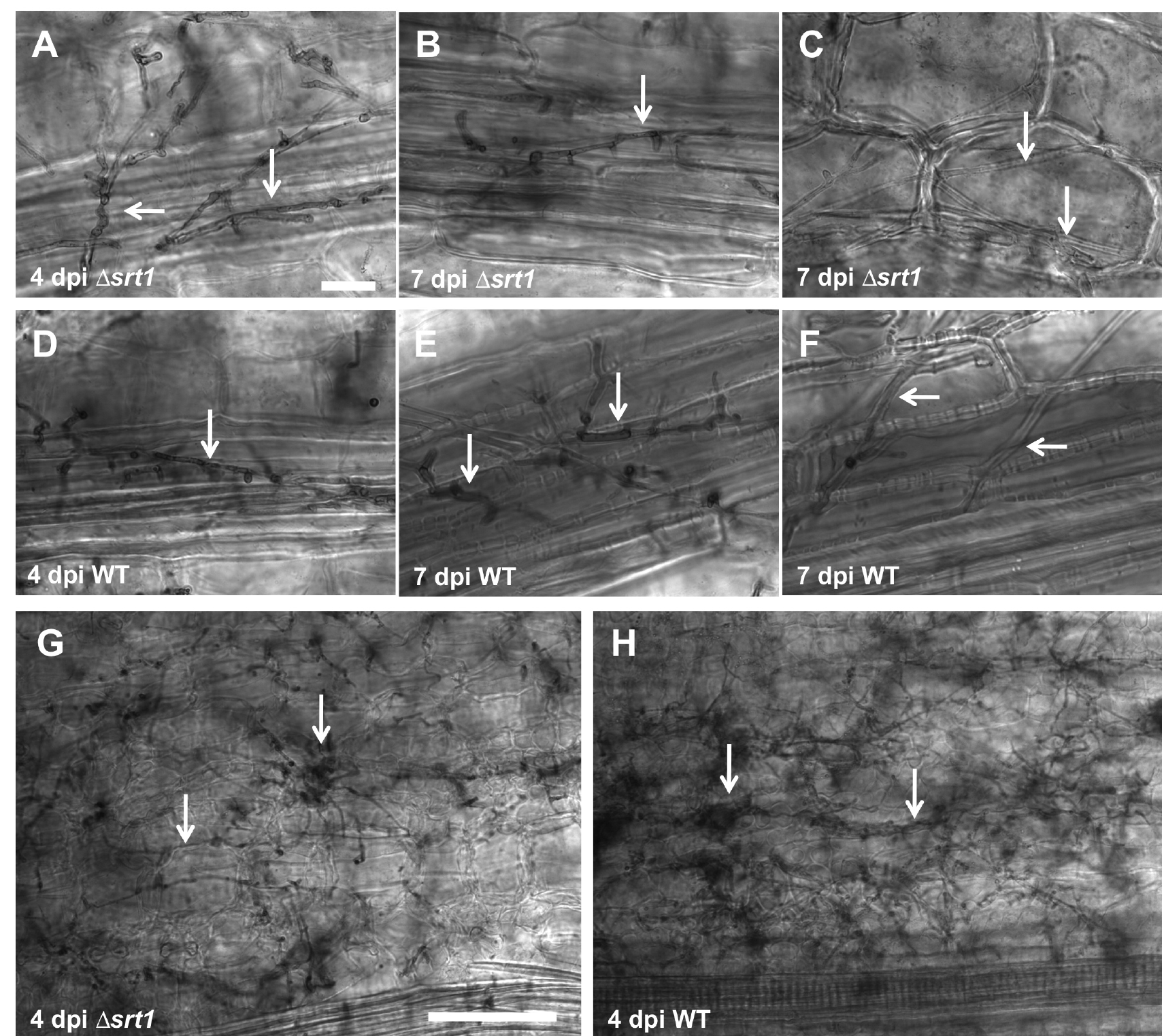

Supplement: Figure S2 — SG200Δ srt1 hyphae do not differ with respect to leaf colonization from SG200 hyphae at 4 and 7 dpi during disease progression. Chlorazole Black E staining of maize leaves infected with SG200Δsrt1 and SG200 wild type at 4 and 7 dpi. (A) and (D) show hyphae of both strains at 4 dpi growing in the vicinity of a vascular bundle. (B) and (E) display hyphae at 7 dpi growing in the vicinity of a vascular bundle. (C) and (F) display collapsed hyphae that appear at 7 dpi in infections with both SG200 and SG200Δsrt1. (G) and (H) show an overview of a larger area infected with the two strains, respectively. In both cases, hyphae spread within the plant leave tissue. Scale bars indicate a magnification of 20 µm for (A), (B), (C), (D), (E), and (F) and 100 µm for (G) and (H). (1.62 MB TIF) [file pbio.1000303.s002.tif]

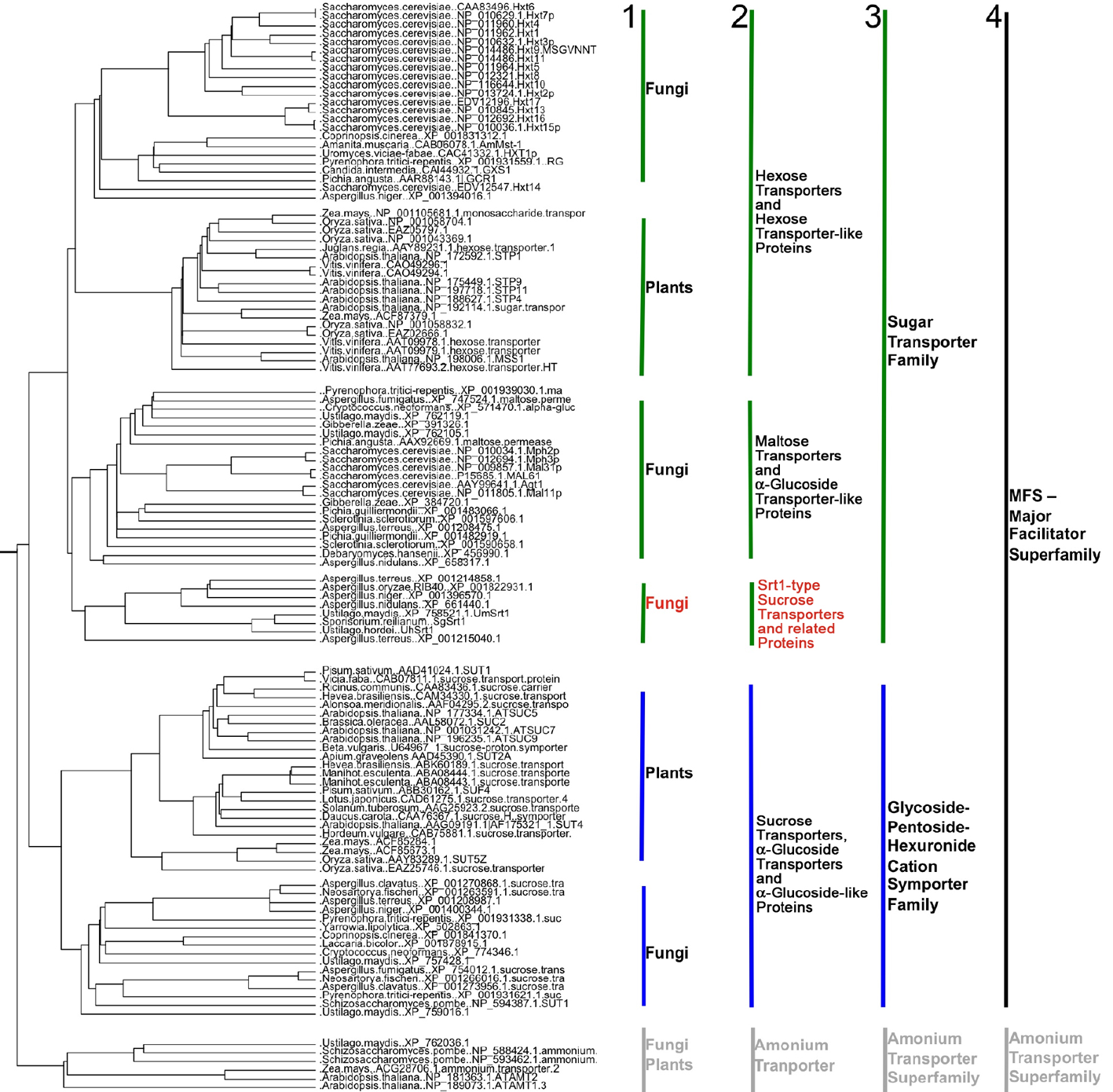

Supplement: Figure S3 — Comparative phylogenetic analyses of Srt1. The Srt1 amino acid sequence was aligned with 117 transporter sequences obtained by BLASTP analysis. The analyses include fungal and plant sequences with the highest similarity to Srt1, fungal and plant sequences with highest homology to A. thaliana sucrose transporters, as well as fungal and plant ammonium transporter sequences as out-group (Table S2). A high phylogenetic distance is observed between the clade of potential plant and fungal sucrose transporters belonging to the Glycoside-Pentoside-Hexuronide Cation Symporter Family and the Srt1-like sucrose transporters belonging to the sugar transporter family. Species names, accession numbers, and where available gene names are given. (0.91 MB TIF) [file pbio.1000303.s003.tif]

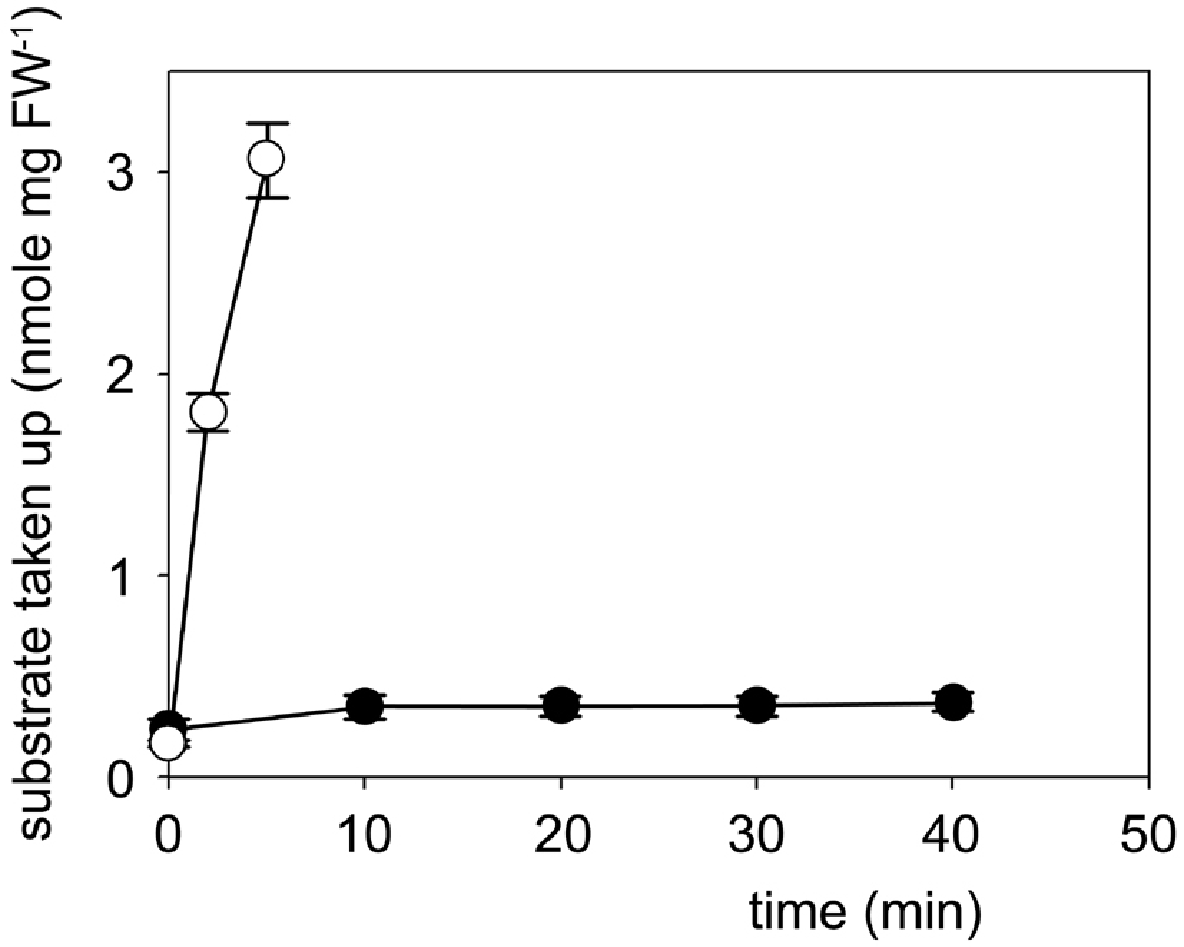

Supplement: Figure S4 — 14C-maltose is not a substrate for Srt1. Uptake of 14C-maltose (closed circles) was determined in parallel with the uptake of 14C-sucrose (open circles) in the same srt1-expressing S. cerevisiae cells that had been used to determine transport in Figure 4. The extracellular pH was 5.0, substrate concentration was 1 mM. Although 14C-maltose transport was analyzed for much longer than the transport of 14C-sucrose (see also Figure 4), no significant import of 14C-maltose into srt1-expressing cells could be observed. Error bars represent standard error (n = 3). (0.07 MB TIF) [file pbio.1000303.s004.tif]

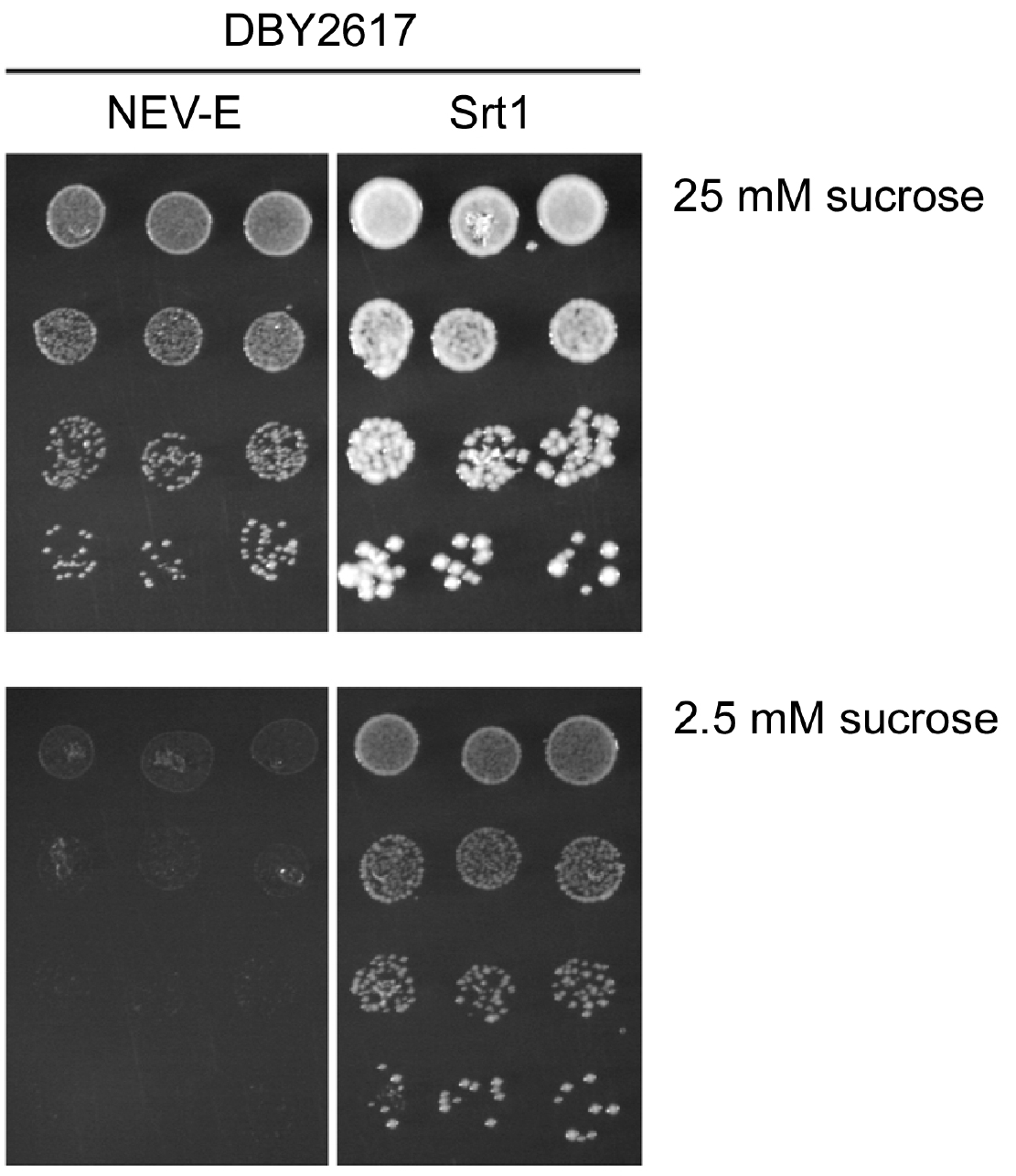

Supplement: Figure S5 — Srt1 complements the growth defect of S. cerevisiae strain DBY2617. DBY2617 possesses a cytoplasmic invertase, but lacks an extracellular invertase and a sucrose transport activity. Therefore, it cannot use extracellular sucrose as carbon source. Transformation with a plasmid that drives expression of srt1 complements this defect and allows growth on sucrose as sole carbon source. Transformation with the empty vector (NEV-E) allows only limited growth that is due to passive diffusion of sucrose into the cells. (0.43 MB TIF) [file pbio.1000303.s005.tif]
