# Supplementary material for: A Novel High-Affinity Sucrose Transporter Is Required for Virulence of the Plant Pathogen Ustilago maydis
Source: PLoS Biol. 2010 Feb 9;8(2):e1000303. doi: 10.1371/journal.pbio.1000303 (PMC2817709; doi:10.1371/journal.pbio.1000303)
Supplement: Table S1 — U. maydis transporter proteins of the Major Facilitator Superfamily. Accession number, gene number (MUMDB [IPR007114 Major facilitator superfamily; http://mips.helmholtz-muenchen.de/genre/proj/ustilago/) and predicted function of the putative transport proteins used to calculate the phylogenetic tree shown in Figure S1. (0.04 MB DOC) [file pbio.1000303.s006.doc]

**Table S1**

**Accession Um-number Annotation**

XP_762630 um10946 nicotinic acid permease

XP_756181 um00034 multidrug-resistance transporter

XP_756208 um00061 quinate transport

XP_756263 um00116 nicotinamide mononucleotide permease

XP_756353 um00206.2 hypothetical protein

XP_756369 um00222.2 monocarboxylate transporter

XP_756521 um00374 allantoate-ureidosuccinate permease

XP_756624 um00477 monocarboxylate transporter

XP_756676 um00529 carboxylic acid transport

XP_756826 um00679 vacuolar basic amino acid transporter

XP_756867 um11171 sugar transport

XP_756947 um00800 inorganic phosphate permease

XP_756962 um00815 multidrug resistant protein

XP_756989 um00842 aflatoxin efflux pump

XP_757198 um01051 multidrug-resistance transporter

XP_757303 um01156 myo-inositol transporter

XP_757577 um11339 siderophore iron transporter

XP_757582 um01435 monocarboxylate transporter

XP_757623 um01476 low-affinity hexose facilitator

XP_757675 um01528 conserved hypothetical protein

XP_757695 um10815 conserved hypothetical protein

XP_757800 um01653 involved in vacuolar protein sorting

XP_757803 um01656 sugar transporter

XP_758015 um01868 glycerophosphoinositol transporter

XP_758029 um01882 multidrug resistance proteins

XP_758046 um01899 multidrug resistance protein

XP_758091 um01944 allantoate permease

XP_758111 um01964 multidrug resistance protein

XP_758129 um01982 aminotriazole resistance protein

XP_758143 um01996 multidrug-resistance transporter

XP_758155 um02008 conserved hypothetical protein

XP_761214 um11773 conserved hypothetical protein

XP_761224 um11777 conserved hypothetical protein

XP_761269 um05122 carboxylic acid transport

XP_762496 um06349 siderophore iron transporter

XP_758184 um02037 hexose transporter

XP_758209 um02062 multidrug resistance protein

XP_758352 um10072 monosaccharide transporter

XP_758521 um02374 Srt1 - sucrose transporter

XP_758637 um02490 multidrug resistance H^+^ antiporter

XP_758732 um02585 vacuolar polyamine-H^+^ antiporter

XP_758745 um02598 multidrug transporter

XP_758763 um12193.2 multidrug resistant protein

XP_758851 um02704 allantoate permease

XP_758870 um02723.2 multidrug-resistance transporter

XP_758952 um10608 quinate permease

XP_759047 um02900 conserved hypothetical protein

XP_759181 um03034 conserved hypothetical protein

XP_759262 um03115 drug resistance protein

XP_759531 um03384 mediator of drug efflux

XP_759620 um03474 conserved hypothetical protein

XP_759654 um03507 conserved hypothetical protein

XP_759701 um03554 conserved hypothetical protein

XP_759759 um03612 conserved hypothetical protein

XP_759766 um03619 multidrug resistant protein

XP_760035 um03888 multidrug resistant protein

XP_760042 um11514 high-affinity glucose transporter

bankit1186173 um11516 conserved hypothetical protein

XP_760055 um03908 vacuolar polyamine-H^+^ antiporter

XP_760685 um04538 allantoate-ureidosuccinate permease

XP_760802 um04655 conserved hypothetical protein

XP_760827 um04680 monocarboxylate transporter

XP_760557 um04410 siderophore iron transporter

XP_760625 um04478 myo-inositol transporter

XP_761170 um05023 monosaccharide transporter

XP_761181 um05034 cephamycin export protein

XP_761781 um15021 conserved hypothetical protein

XP_761033 um04886 related to HOL1 protein

XP_761589 um05442 monocarboxylate transporter

XP_761599 um05452.2 polyamine transport protein

XP_761749 um05602 sugar transporter

XP_761395 um05248 polyamine transport protein

XP_761407 um05260 inorganic phosphate transporter

XP_761540 um05393 multidrug resistant protein

XP_761543 um05396.2 conserved hypothetical protein

XP_761558 um05411 quinate permease

XP_761561 um05414 aflatoxin efflux pump

XP_761568 um05421 multidrug resistance protein

XP_762105 um05958 maltose permease

XP_762119 um05972 maltose permease

XP_762128 um05981 vacuolar polyamine-H^+^ antiporter

XP_762223 um06076 quinate transport protein

XP_762538 um06391 multidrug resistant protein

XP_762637 um06490 inorganic phosphate permease

XP_757428 um01281 alpha-glucoside permease

XP_759016 um02869 alpha-glucoside permease

XP_762036.1 um05889 high affinity ammonium transporter

XP_760670 um04523 Ump1 ammonium transporter

**Table S1** Accession numbers, Um-numbers [MUMDB (IPR007114  Major facilitator superfamily; <http://mips.gsf.de/genre/proj/ustilago/>)] and predicted functions of the putative transport proteins used to calculate the phylogenetic tree shown in Figure S1.
