# Supplementary material for: A Novel High-Affinity Sucrose Transporter Is Required for Virulence of the Plant Pathogen Ustilago maydis
Source: PLoS Biol. 2010 Feb 9;8(2):e1000303. doi: 10.1371/journal.pbio.1000303 (PMC2817709; doi:10.1371/journal.pbio.1000303)
Supplement: Table S2 — Proteins used for comparative phylogenetic analyses of Srt1. Accession numbers, putative or determined functions of the transport proteins used to calculate the phylogenetic tree shown in Figure S3. (0.06 MB DOC) [file pbio.1000303.s007.doc]

**Supplementary Table S2**

**Accession Annotation Species**

AAG09191/Q9FE59 AtSUC4 *Arabidopsis thaliana*

NP_001031242.1 AtSUC7 *Arabidopsis thaliana*

NP_175449.1 STP9 *Arabidopsis thaliana*

NP_177334.1 AtSUC5 *Arabidopsis thaliana*

NP_181363.1 AtAMT2 *Arabidopsis thaliana*

NP_189073.1 AtAMT1;3 *Arabidopsis thaliana*

NP_192114.1 sugar transporter *Arabidopsis thaliana*

NP_196235.1 AtSUC9 *Arabidopsis thaliana*

NP_198006.1 MSS1 *Arabidopsis thaliana*

XP_001270868.1 sucrose transporter *Aspergillus clavatus*

XP_001273956.1 sucrose transporter *Aspergillus clavatus*

XP_747524.1 maltose permease *Aspergillus fumigatus*

XP_754012.1 sucrose transporter *Aspergillus fumigatus*

XP_658317.1 hypothetical protein *Aspergillus nidulans*

XP_661440.1 hypothetical protein *Aspergillus nidulans*

XP_001394016.1 hypothetical protein *Aspergillus niger*

XP_001396570.1 hypothetical protein *Aspergillus niger*

XP_001400344.1 hypothetical protein *Aspergillus niger*

XP_001822931.1 hypothetical protein *Aspergillus oryzae*

XP_001208475.1 hypothetical protein *Aspergillus terreus*

XP_001208987.1 hypothetical protein *Aspergillus terreus*

XP_001214858.1 hypothetical protein *Aspergillus terreus*

XP_001215040.1 hypothetical protein *Aspergillus terreus*

XP_001831312.1 hypothetical protein *Coprinopsis cinerea*

XP_001841370.1 hypothetical protein *Coprinopsis cinerea*

XP_571470.1 a-glucoside transporter *Cryptococcus neoformans*

XP_774346.1 hypothetical protein *Cryptococcus neoformans*

XP_456990.1 hypothetical protein *Debaryomyces hansenii*

XP_384720.1 hypothetical protein *Gibberella zeae*

XP_391326.1 hypothetical protein *Gibberella zeae*

ABK60189.1 sucrose transporter 5 *Hevea brasiliensis*

XP_001878915.1 hypothetical protein *Laccaria bicolor*

ABA08443.1 SUC4-type transporter *Manihot esculenta*

XP_001263591.1 sucrose transporter *Neosartorya fischeri*

XP_001266016.1 sucrose transporter *Neosartorya fischeri*

AAY83289.1 SUT5Z *Oryza sativa*

EAZ25746.1 sucrose transporter *Oryza sativa*

EAZ02666.1 hypothetical protein *Oryza sativa*

NP_001043369.1 hypothetical protein *Oryza sativa*

NP_001058704.1 hypothetical protein *Oryza sativa*

NP_001058832.1 hypothetical protein *Oryza sativa*

EAZ05797.1 hypothetical protein *Oryza sativa*

AAX92669.1 maltose permease *Pichia angusta*

XP_001483066.1 hypothetical protein *Pichia guilliermondii*

XP_001482919.1 hypothetical protein *Pichia guilliermondii*

XP_001931338.1 SUC2 *Pyrenophora tritici-rep.*

XP_001931621.1 sucrose transporter *Pyrenophora tritici-rep.*

XP_001939030.1 maltose permease *Pyrenophora tritici-rep.*

AAY99641.1 Agt1 *Saccharomyces cerevisiae*

NP_009857.1 Mal31p *Saccharomyces cerevisiae*

NP_010034.1 Mph2p *Saccharomyces cerevisiae*

NP_011805.1 Mal11p *Saccharomyces cerevisiae*

NP_012694.1 Mph3p *Saccharomyces cerevisiae*

P15685.1 MAL61 *Saccharomyces cerevisiae*

NP_588424.1 Amt1 *Schizosaccharomyces pombe*

NP_593462.1 Amt2 *Schizosaccharomyces pombe*

NP_594387.1 Sut1 *Schizosaccharomyces pombe*

XP_001590658.1 hypothetical protein *Sclerotinia sclerotiorum*

XP_001597606.1 hypothetical protein *Sclerotinia sclerotiorum*

FJ754644 SgSrt1 *Sporisorium reilianum*

FJ754645 UhSrt1 *Ustilago hordei*

XP_757428.1 hypothetical protein *Ustilago maydis*

XP_758521.1 UmSrt1 *Ustilago maydis*

XP_759016.1 hypothetical protein *Ustilago maydis*

XP_762036.1 hypothetical protein *Ustilago maydis*

XP_762105.1 hypothetical protein *Ustilago maydis*

XP_762119.1 hypothetical protein *Ustilago maydis*

AAT09978.1 hexose transporter *Vitis vinifera*

AAT09979.1 hexose transporter *Vitis vinifera*

AAT77693.2 hexose transporter HT2 *Vitis vinifera*

CAO49294.1 hypothetical protein *Vitis vinifera*

CAO49296.1 hypothetical protein *Vitis vinifera*

XP_502863.1 hypothetical protein *Yarrowia lipolytica*

ACF85284.1 hypothetical protein *Zea mays*

ACF85673.1 hypothetical protein *Zea mays*

ACG28706.1 ammonium transporter 2 *Zea mays*

ACF87379.1 hypothetical protein *Zea mays*

NP_001105681.1 MST 1 *Zea mays*

EDV12547 Hxt14 *Saccharomyces cerevisiae*

NP_012321 Hxt8 *Saccharomyces cerevisiae*

NP_014486 Hxt9 *Saccharomyces cerevisiae*

NP_014486 Hxt11 *Saccharomyces cerevisiae*

CAA83496 Hxt6 *Saccharomyces cerevisiae*

EDV12196 Hxt17 *Saccharomyces cerevisiae*

NP_012692 Hxt16 *Saccharomyces cerevisiae*

NP_116644 Hxt10 *Saccharomyces cerevisiae*

NP_010845 Hxt13 *Saccharomyces cerevisiae*

NP_011964 Hxt5 *Saccharomyces cerevisiae*

NP_011962 Hxt1 *Saccharomyces cerevisiae*

NP_011960 Hxt4 *Saccharomyces cerevisiae*

NP_010629.1 Hxt7 *Saccharomyces cerevisiae*

NP_013724.1 Hxt2 *Saccharomyces cerevisiae*

NP_010632.1 Hxt3 *Saccharomyces cerevisiae*

NP_010036.1 Hxt15 *Saccharomyces cerevisiae*

CAB06078.1 AmMst-1 *Amanita muscaria*

CAC41332.1 HXT1 *Uromyces viciae-fabae*

XP_001931559.1 RGT2 *Pyrenophora tritici-rep.*

CAI44932.1 GXS1 *Candida intermedia*

AAR88143.1 GCR1 *Pichia angusta*

AAY89231.1 hexose transporter 1 *Juglans regia*

NP_172592.1 STP1 *Arabidopsis thaliana*

NP_197718.1 STP11 *Arabidopsis thaliana*

NP_188627.1 STP4 *Arabidopsis thaliana*

ABB30162.1 SUF4 *Pisum sativum*

AAG25923.2 sucrose transporter 4 *Solanum tuberosum*

CAD61275.1 sucrose transporter 4 *Lotus japonicus*

CAA76367.1 sucrose/H^+^ symporter *Daucus carota*

ABA08444.1 sucrose transporter 4 *Manihot esculenta*

CAB75881.1 sucrose transporter 2 *Hordeum vulgare*

AAD41024.1 SUT1 *Pisum sativum*

CAB07811.1 sucrose transporter *Vicia faba*

CAA83436.1 sucrose carrier *Ricinus communis*

U64967_1 sucrose transporter *Beta vulgaris*

AAL58072.1 SUC2 *Brassica oleracea*

AAF04295.2 sucrose transporter 1 *Alonsoa meridionalis*

CAM34330.1 sucrose transporter 6 *Hevea brasiliensis*

AAD45390.1 SUT2A *Apium graveolens*

**Table S2** Accession numbers, putative or determined functions of the transport proteins used to calculate the phylogenetic tree shown in Figure S3.
